# Microglia-Specific Promoter Activities of *HEXB* Gene

**DOI:** 10.3389/fncel.2022.808598

**Published:** 2022-03-10

**Authors:** Sahil Shah, Lilly M. Wong, Kendra Ellis, Brittany Bodnar, Sami Saribas, Julia Ting, Zhengyu Wei, Yuyang Tang, Xianwei Wang, Hong Wang, Binhua Ling, David M. Margolis, J. Victor Garcia, Wenhui Hu, Guochun Jiang

**Affiliations:** ^1^Center for Metabolic Disease Research, Temple University Lewis Katz School of Medicine, Philadelphia, PA, United States; ^2^Department of Pathology and Laboratory Medicine, Temple University Lewis Katz School of Medicine, Philadelphia, PA, United States; ^3^University of North Carolina HIV Cure Center, The University of North Carolina at Chapel Hill, Chapel Hill, NC, United States; ^4^Southwest National Primate Research Center, Host-Pathogen Interaction Program, Texas Biomedical Research Institute, San Antonio, TX, United States; ^5^Department of Medicine, Microbiology and Immunology, Epidemiology, The University of North Carolina at Chapel Hill, Chapel Hill, NC, United States; ^6^International Center for the Advancement of Translational Science, Division of Infectious Diseases, UNC Center for AIDS Research, University of North Carolina at Chapel Hill, Chapel Hill, NC, United States; ^7^Department of Biochemistry and Biophysics, The University of North Carolina at Chapel Hill, Chapel Hill, NC, United States

**Keywords:** *HexB*, *CD68*, microglia, astrocytes, gene therapy, gene editing

## Abstract

Adeno-associated virus (AAV)-mediated genetic targeting of microglia remains a challenge. Overcoming this hurdle is essential for gene editing in the central nervous system (CNS). Here, we characterized the minimal/native promoter of the *HEXB* gene, which is known to be specifically and stably expressed in the microglia during homeostatic and pathological conditions. Dual reporter and serial deletion assays identified the critical role of the natural 5’ untranslated region (−97 bp related to the first ATG) in driving transcriptional activity of the mouse *Hexb* gene. The native promoter region of mouse, human, and monkey *HEXB* are located at −135, −134, and −170 bp to the first ATG, respectively. These promoters were highly active and specific in microglia with strong cross-species transcriptional activities, but did not exhibit activity in primary astrocytes. In addition, we identified a 135 bp promoter of *CD68* gene that was highly active in microglia but not in astrocytes. Considering that *HEXB* is specifically expressed in microglia, these data suggest that the newly characterized microglia-specific *HEXB* minimal/native promoter can be an ideal candidate for microglia-targeting AAV gene therapy in the CNS.

## Introduction

Adeno-associated virus (AAV) delivery of gene editors is one of the foremost technologies in development for gene therapy of central nervous diseases (CNS), including human immunodeficiency virus (HIV) infection. Finding an effective gene delivery system is essential. One way is to find an effective promoter and express the selected gene editor specifically in the brain microglia. Considering the size restrictions for AAV packaging, it is important to identify a promoter that is both small and transcriptionally active. Microglia-specific gene promoters have been studied in the mouse microglia cell models using mouse myeloid cell-specific promoter including the *CD68* promoter (Kettenmann et al., 2011; Rosario et al., 2016); however, it is not clear whether these promoters are effective in human primary microglial cells. Since CD68 is expressed in both microglia and macrophages, it may not be specific to microglia in the human brain.

Recent RNAscope and single-cell RNA sequencing (scRNA-seq) analysis determined that *Hexb* is exclusively expressed in brain microglia but not in monocytes/macrophages, with a much lower expression in other neural cells in the CNS (Hickman et al., 2013; Masuda et al., 2020). Importantly, this newly defined microglia-specific gene retains its expression even under various pathological conditions while many other microglia core genes are substantially downregulated (Hickman et al., 2013; Masuda et al., 2020). This indicates that *HEXB* could be an excellent option for microglia-specific targeting gene therapy for CNS injury and diseases.

In this study, we determined whether *HEXB* promoter is specific to microglia and whether its activity is species dependent *in vitro*. We also optimized the essential element of the native human *HEXB* promoter that has the potential to be used in the AAV gene therapy for brain HIV eradication. For an efficient gene therapy, it requires a small gene insert to meet requirements for packaging capacity and transduction efficiency of AAV delivery into the brain. Therefore, characterization of a proper microglia-specific promoter, such as *HEXB*, is important for the development of gene editing tools specific for neurological diseases in the future.

## Materials and Methods

### Construction of *HEXB* Promoter Vectors

NEBuilder^®^ *HiFi* DNA Assembly cloning kit (NEB, Cat# E5520S) was used to clone *HEXB* gene promoters of various sizes and species into a promoterless AAV vector (TP1768) containing *gaussia* dura luciferase (gdLuc) and green fluorescent protein (GFP) dual reporter (LG). PCR from genomic DNA was performed to generate *HEXB* gene promoter inserts using Phusion High-Fidelity PCR Master Mix kit (Thermo Fisher, Waltham, MA, United States, F531). The three main sources of promoter DNA were human induced pluripotent stem cells (iPS), mouse embryonic stem cells (ESC), and monkey LC30 cells. The original AAV vector pX601-AAV-CMV::NLS-SaCas9-NLS-3xHA-bGHpA;U6::BsaI-sgRNA (Addgene plasmid # 61591, a gift from Feng Zhang) was used to generate the mHexB-330ΔUTR-LG (TP1759) at *Xho*I/*EcoR*I sites using two PCR products from mouse ESC genome and LG expressing plasmid. An oligonucleotide fragment containing multiple cloning sites (MCS) was cloned into TP1759 *via Xba*I/*Xho*I to generate promoterless AAV-MCS-LG vector (TP1768). Then, various sizes of PCR products for HexB promoters were cloned *via Xha*I/*BamH*I sites in TP1768 to generate all HexB vectors. The PCR of hCD68 promoter (695 and 135 bp) from pAAV CD68-hM4D(Gi)-mCherry (Addgene plasmid # 75033, a gift from Bryan Roth) was cloned into TP1768 *via EcoR*V/*BamH*I sites. All PCR products were purified from agarose gel, and the digestion product was purified using Monarch PCR and DNA Cleanup Kit (NEB, T1030S). The backbone vector and the insert were ligated with the NEB HiFi assembly system, using a 1:10 ratio of backbone to insert. The CMV-driven LG vector was generated *via Mlu*I/*BamH*I sites of TP1759 using standard T4 ligation kit. The ligation was then transformed into NEB stable competent cells and the plate was incubated overnight at 30°C. In creating most vectors, colony PCR was used to confirm that the correct promoter size was inserted prior to sequencing. The correct clones were verified by restriction enzyme digestion and Sanger sequencing as well as functional measures.

### Functional Testing of *HEXB* Gene Promoter in Cell Culture

The initial experiments to determine the activity of the *HEXB* promoters were performed in HEK293T cells using standard PeiMax transfection method. Then the promoter activities were tested in C20 human microglia and SIM-A9 mouse microglia cell lines using Glial-Mag kit (OZ Biosciences; San Diego, CA, United States, Cat # GL002500). The cells were cultured under standard conditions and seeded into 96-well plates at a density of 3.0 × 10^4^/100 μl. HEK293T cells were cultured in DMEM with 10% FBS. C20 cells were cultured in a DMEM with 5% FBS medium. SIM-A9 cells were cultured in DMEM with 10% FBS and 5% horse serum. After growing overnight, the cells were transfected with indicated AAV-*HEXB* vectors. Transfections were completed in quadruplicates where each well received 100 ng of promoter reporter DNA and 20 ng of pSEAP2 control vector for transfection normalization. Empty promoterless LG vector was used as a negative control (empty vector). The CMV-driven pAAV-Flag-LG or pcDNA3-GFP vectors were used as the positive controls with transfection efficiency at 80–90% in HEK293T cells and 30–40% in C20 or SIM-A9 cell lines. At 24–96 h post-transfection, the cells were assessed for expression *via* fluorescence microscopy of GFP and gaussia luciferase assay.

Primary human microglial cells were purchased from (Celprogen, Torrance, CA, United States) while human primary astrocytes were obtained from (ScienCell Research Laboratories, Carlsbad, CA, United States). Primary rhesus macaque microglia and astrocytes were isolated from fresh animal brains with purities > 99%. Microglia were cultured in DMEM/F12 (Gibco, Waltham, MA, United States) supplemented with 10% defined fetal bovine serum (Cytiva Life Sciences, Marlborough, MA, United States), 10 ng/ml recombinant human M-CSF (R&D Systems, Minneapolis, MN, United States), 100 μg/mL streptomycin, 100 U/mL penicillin, 2 mM L-glutamine, 3 mM sodium pyruvate, and 10 mM HEPES. Astrocytes were cultured in astrocyte medium (AM, ScienCell Research Laboratories, Carlsbad, CA, United States) supplemented with FBS, P/S, and Astrocyte Growth Supplement (AGS) (ScienCell Research Laboratories, Carlsbad, CA, United States). All cells were cultured in 37°C incubator with 5% CO_2_. Cell transfections were carried out in 96-well plate with 1.0 × 10^4^ target cells where each well received 100 ng DNA with a mix of Lipofectamine 3000 (Invitrogen, Waltham, MA, United States) in Opti-MEM.

### Luciferase Assays and Green Fluorescent Protein Microscopy

Approximately 50 μl of culture supernatants were harvested 24–72 h post-transfection. Prior to gaussia luciferase assay, supernatant from cells was combined with an equal volume of coelenterazine (CTZ) substrate that was diluted 1:50 with CTZ dilution buffer. Prior to measurement, samples were allowed to incubate for 5 min at room temperature. Luciferase activity was measured by bioluminescence plate reader (5 s integration) per manufacturer’s instructions (Nanolight Technology, Pinetop, AZ, United States). SEAP activity in the culture supernatants were measured with QUANTI-Blue™ Solution (InvivoGen, San Diego, CA, United States) following the manufacturer’s protocol. The data were analyzed in comparison with control empty vector transfection after SEAP normalization. At the end of transfection, the GFP+ cells were also analyzed by fluorescence microscopy to determine the gene transcription driven by promoters.

### Flow Cytometry

Twenty four to seventy two hours post-transfection, 1-2 × 10^5^ cells were collected and washed once with cold PBS by centrifugation for 5 min at 300 × *g*. Cells were fixed in buffered 1% formaldehyde solution, which were subjected for flow cytometry analysis to measure GFP expression and determine the transfection efficiency. The data were analyzed by Software FlowJo™ v10.8 Software (BD Life Sciences, Franklin Lakes, NJ, United States). Cell viability was evaluated using dye LIVE/DEAD Fixable Far Red stain (Invitrogen) (Life Technologies, Carlsbad, CA, United States) during flow cytometry.

### Statistical Analysis

Quantification of fold changes in promoter groups compared with corresponding promoterless or promoter groups was performed using excel software. Statistical analysis was performed using (Prism GraphPAD Software, San Diego, CA, United States). Significance was determined by two-tailed student’s *t*-test between two groups or by one-way ANOVA for multiple comparisons, at *p < 0.05; ^**^p < 0.01; ^***^p < 0.001 and ^****^p < 0.0001. Data were presented as mean ± SE. The size and type of individual samples were specified in the figure legends.

## Results

### Validation of Mouse *Hexb* Gene Promoter Activity With Dual Reporter Assay

**FIGURE 1:**
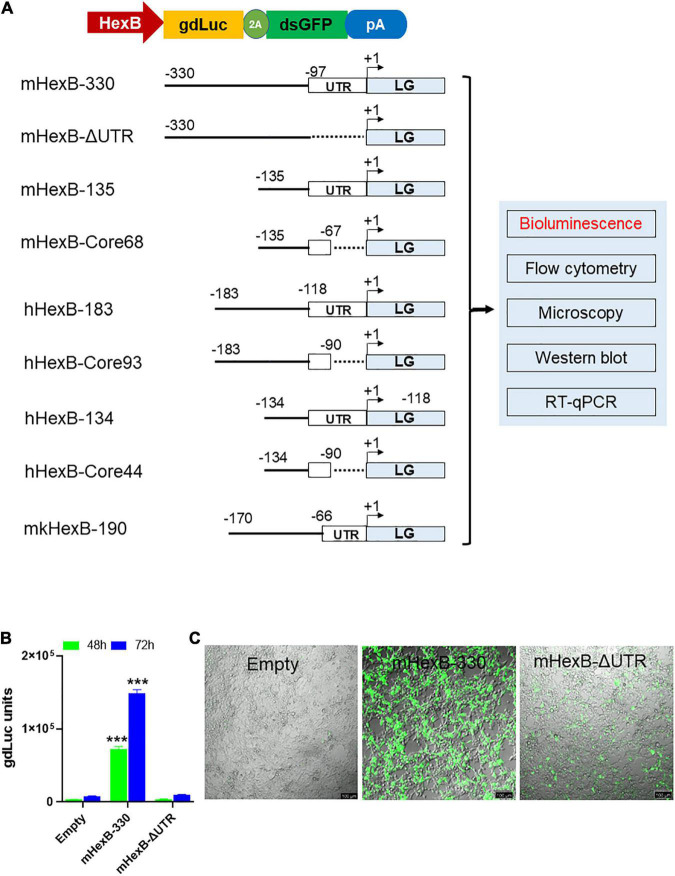
LG dual reporter assays to measure *HEXB* promoter activity. **(A)** Diagram for LG dual reporter assay driven by *HEXB* promoters derived from mouse, human, or monkey cells. **(B)** HEK293T cells were transfected with mouse 330 bp *Hexb* promoter-driven dual LG reporter (mHexb-330) and no *Hexb* 5’UTR-driven dual LG reporter (mHexB-no UTR, −330 to −97) plasmid in 96-well plate, where Empty promoterless LG reporter vector served as negative control. At 48–72 h after transfection, gdLuc levels in culture media were measured to determine the *Hexb* promoter activities. **(C)** Representative images were taken to show GFP expression in HEK293T cells 72 h after transfection (scale bar, 100 μm). ***p < 0.001 analyzed with student’s *t*-test compared with empty vector transfection control (*n* = 4).

We established dual reporter assay using gdLuc and GFP expression (thereafter referred as LG) that offer several benefits. It can measure the dynamic quantification of secreted luciferase in culture media with highly sensitive gdLuc assay. The transcriptional activity can also be determined by GFP expression with either fluorescence microscopy or flow cytometry (Figure 1A). Both measures are useful for a fast and high-throughput analysis of *HEXB* promoter activity. Based on previous reports on the promoter activity of the mouse *Hexb* gene (Norflus et al., 1996; Urbanelli et al., 2014), we initially evaluated the activity of *Hexb* promoter region −330 bp upstream of ATG (mHexB-330) (Figure 1A). We selected HEK293T cells as the test platform because of their high transfection efficiency. We found that the mHexB-330 facilitated robust gdLuc reporter expression by 20∼22 fold 48 h after transfection (Figures 1B,C), which is consistent with a previous report using chloramphenicol acetyltransferase reporter assay in NIH-3T3 cells (Norflus et al., 1996). Fluorescent microcopy further confirmed the dramatic increase in GFP expression in mHexB-330 transfected cells (Figure 1C). When the 5’-UTR region was deleted, no transcriptional activity was observed, which validated the critical role of 5’-UTR (−97) in driving the promoter activity of HexB-330 (Figures 1A,B; Norflus et al., 1996).

### Cloning and Characterization of Essential Promoter Regions for Human and Rhesus Macaque *HEXB*

**FIGURE 2:**
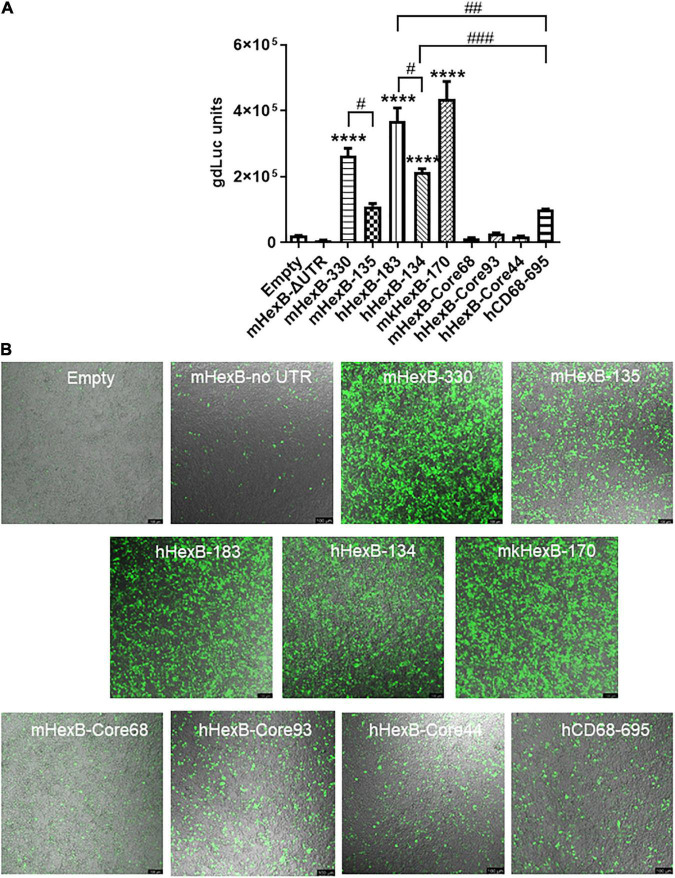
Activity of microglia-related promoters, including *HEXB* and *CD68*, in the induction of gene transcription in HEK293T cells. **(A)** Different sizes of mouse, human, or monkey *HEXB* promoter-driven dual-LG reporters were transfected into HEK293T cells in 96-well plate. At 72 h after transfection, gdLuc levels in culture media were measured to determine the *HEXB* promoter activity. Cells transfected with plasmids driven by other microglia-related promoters, such as *CD68*, were also included, and transcription activity were determined by measuring luciferase levels in culture supernatants. **(B)** Representative images after transfection are shown (scale bar, 100 μm). ****p < 0.0001, compared with empty vector transfection controls (One-way ANOVA, *n* = 4). #*p* < 0.05; ##*p* < 0.01; ###*p* < 0.001, compared with indicated groups (*t*-test, *n* = 4). m, mouse; h, human; mk, monkey.

Because of the high level of sequence conservation across species in the promoter region of *HEXB* genes (Bapat et al., 1988), we cloned the essential/minimal promoter region of human *HEXB* for the potential application in proviral HIV eradication. As expected, the shorter mouse promoter mHexB-135 (135 bp) retained activity (Norflus et al., 1996; Urbanelli et al., 2014), yet it was moderately weaker than mHexB-330 (Figures 1A, 2). Similarly, both human 134 bp *HEXB* (hHexB-134) and 183 bp *HEXB* (hHexB183) promoters have robust promoter activity (Norflus et al., 1996; Urbanelli et al., 2014), while the shorter promoter (HexB-134) retained high activity in driving gene transcription (Figure 2). These essential/minimal regions contain a core region as demonstrated previously in the human fibroblast model (Norflus et al., 1996; Urbanelli et al., 2014). However, the core region alone, mHexB-Core68 (−135 to −67), hHexB-Core93 (−183 to −90), and HexB-core44 (−134 to 90), had no promoter activity (Figure 2), further validating the essential role of the 5’-UTR (Figure 1A; Norflus et al., 1996). To expand the potential application to SIV gene therapy in rhesus macaques, we cloned rhesus macaque 170 bp *HEXB* (mkHexB-170) promoter (Figure 2), which showed similar activity to the human HexB-183 promoter. These data indicate that the essential and minimal/native *HEXB* promoters are sufficient to drive gene transcription.

### *HEXB* Promoter Is Active in Microglia Cell Lines Derived From Different Species

**FIGURE 3:**
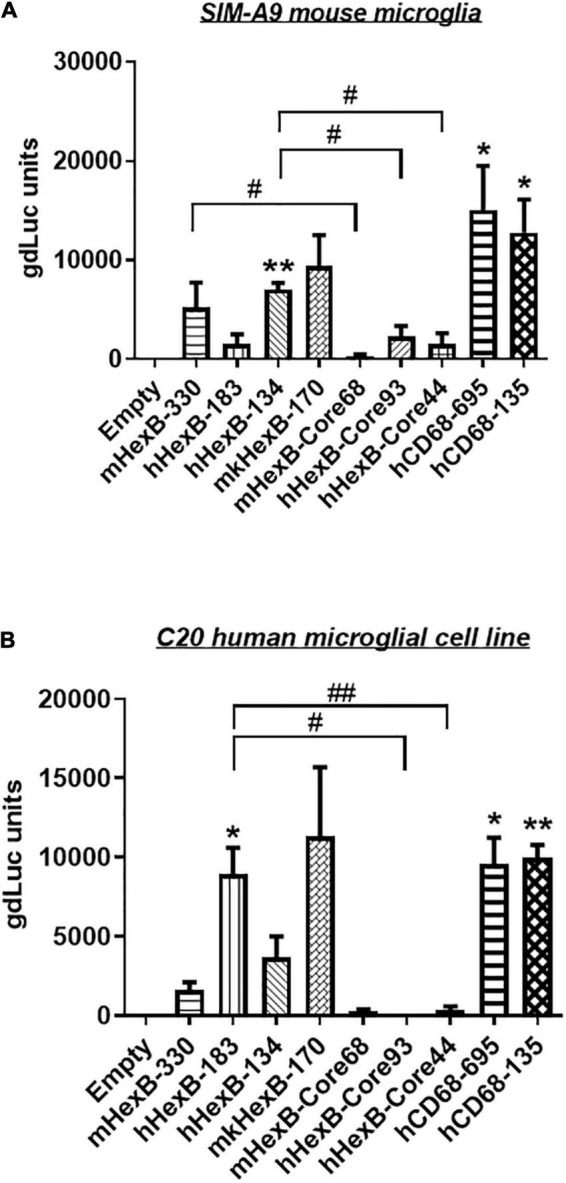
Transcription activity of *HEXB* promoters in mouse SIM-A9 and human C20 microglia cells. *HEXB* promoter-driven dual LG reporters were transfected into mouse SIM-A9 **(A)** or human C20 **(B)** microglial cells. Supernatants were collected 72 h later and subjected to luciferase assays. Luciferase levels were normalized to empty vector, *p < 0.05; **p < 0.01; compared with empty vector transfection control (One-way ANOVA, *n* = 4). #*p* < 0.05; ##*p* < 0.01, compared with cells with indicated groups (*t*-test, *n* = 4). m, mouse; h, human; mk, monkey.

*Hexb* is exclusively expressed in microglia in mouse CNS *in vivo* (Hickman et al., 2013; Masuda et al., 2020). Thus, we determined whether mouse *Hexb* promoter is active in a microglia cell line derived from mouse brain. After transfection, we found that mHexB-330 promoter increased luciferase expression in SIM-A9 mouse microglial cell line (Figure 3A). hHexB-134 and mkHexB-170, was able to drive gene expression in mouse microglia cell line. Surprisingly, the longer human *HEXB* promoter, hHexB-183 was less effective in the activation of transcription. The core promoters without 5’-UTR had weak transcriptional activity.

To see whether there is cross-species activity of *HEXB* promoters, we transfected these reporters into human microglia cell line C20. As shown in Figure 3B, mouse *Hexb* promoters (mHexB-330) had moderate transcriptional capacity. In contrast, both human (hHexB-183) and monkey *HEXB* (mkHexB-170) promoters highly induced transcriptional activity. These data showed that human and rhesus macaque *HEXB* promoters are active in driving gene transcription in both of the microglial cell lines originated from different species, suggesting that *HEXB* promoter activity retains a high level of cross-species interaction. It was interesting that the transcription was similarly driven by human or rhesus macaque *HEXB* promoters, this may reflect the similar genetic background between human and rhesus macaque.

### *HEXB* Native/Minimal Promoter Is Highly Active in Driving Gene Transcription in Human and Rhesus Macaque Primary Microglia Cells, but Not in Primary Astrocytes

**FIGURE 4:**
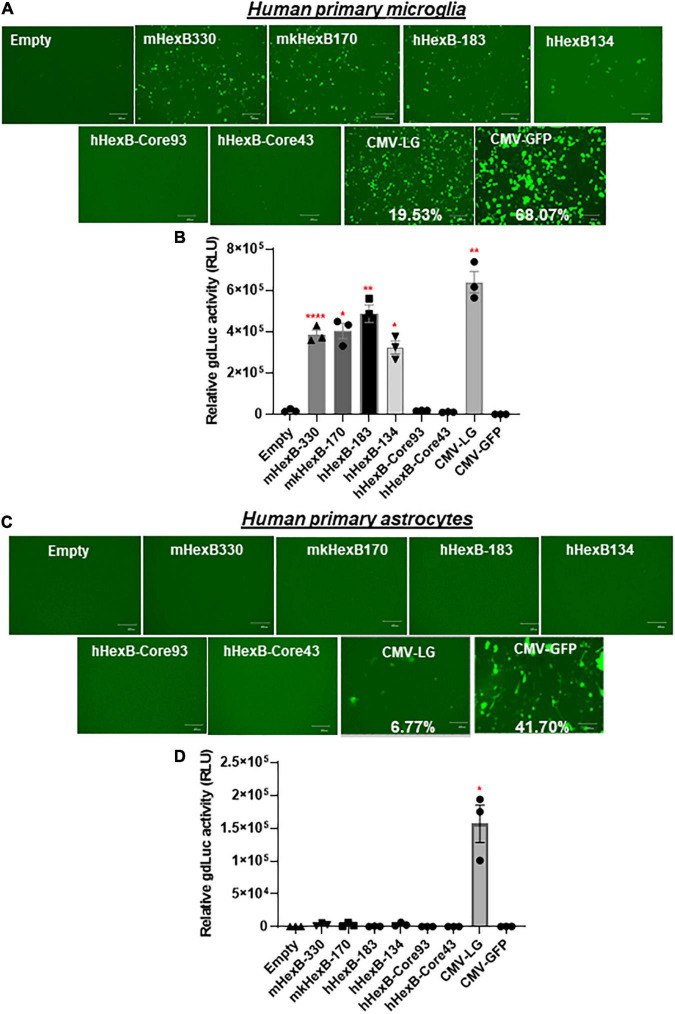
*HEXB* promoter induces reporter transcription mainly in human microglia and not in astrocytes. Native mouse, human, or monkey *HEXB* promoter-driven dual-LG reporters were transfected into human primary microglia **(A,B)** or astrocytes **(C,D)** plated in 96-well plates. After transfection, the presence of GFP+ cells was determined by fluorescence microscopy **(A,C)** where the transfection efficiency was determined by flow cytometry (CMV-LG or CMV-GFP). gdLuc levels **(B,D)** in culture media were used to determine the activity of the indicated *HEXB* promoters. Scale bar, 400 μm. *p < 0.05; **p < 0.01; ****p < 0.0001, compared with empty vector transfection controls (Two tailed *t*-test, *n* = 3). m, mouse; h, human; mk, monkey.

**FIGURE 5:**
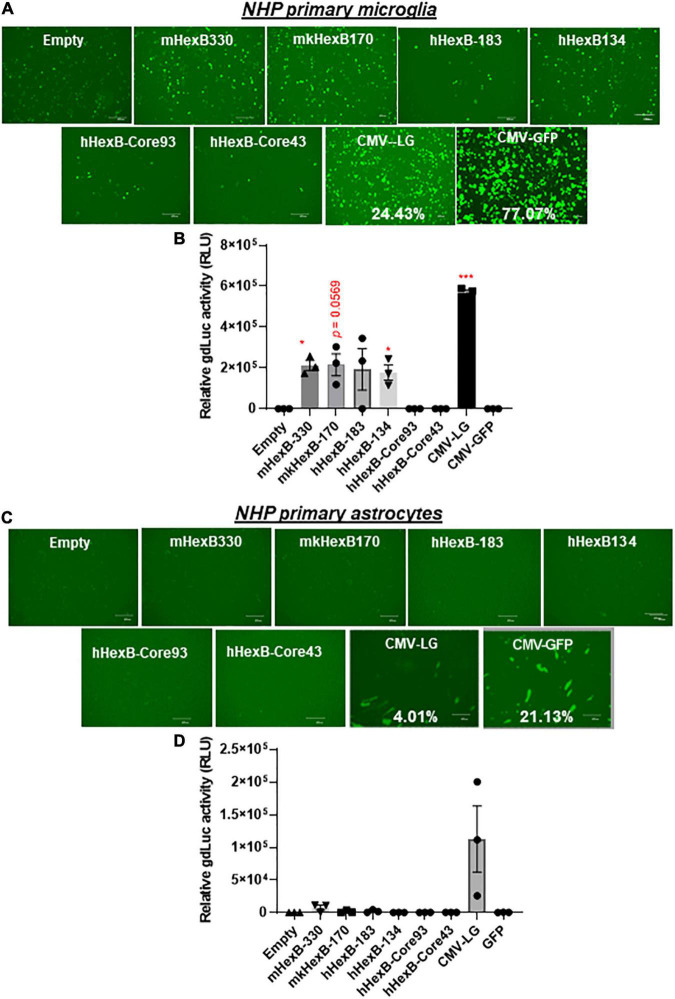
*HEXB* promoter induces reporter transcription mainly in NHP microglia and not in astrocytes. Native or core mouse, human, or monkey *HEXB* promoter-driven dual-LG reporters were transfected into rhesus macaque primary microglia **(A,B)** or astrocytes **(C,D)** plated in 96-well plates. After transfection, the presence of GFP+ cells was determined by fluorescence microscopy **(A,C)** where the transfection efficiency was determined by flow cytometry (CMV-LG or CMV-GFP). The gdLuc levels **(B,D)** in culture media were used to determine the activity of the indicated *HEXB* promoters. Scale bar, 400 μm. *p < 0.05; ***p < 0.001, compared with empty vector transfection controls (Two tailed *T*-test, *n* = 3). m, mouse; h, human; mk, monkey.

While SIM-A9 was derived from cortical tissues collected from postnatal mouse pups and spontaneously immortalized weeks after *in vitro* passaging (ATCC) (Dave et al., 2020), C20 microglia cell line model was originated from primary human microglia cells transformed by SV40 T antigen and human TERT(Rai et al., 2020). In order to generate an AAV vector for microglia targeting in a preclinical or clinical setting in the future, it is essential to test its transcriptional activity in the primary human microglia, but not in transformed human microglial cell lines. Thus, we examined the promoter activities of these *HEXB* promoters in primary microglial cells. We first transfected *HEXB* promoter-driven dual LG reporters into the primary human microglial cells, where a CMV-driven pcDNA3-GFP (CMV-GFP) and CMV-driven dual LG plasmids (CMV-LG) were included as positive transfection controls. We found that the transfection efficiency was relatively high (19.5% in CMV-LG transfected cells and 68% in CMV-GFP plasmids) while all these *HEXB* promoters highly induced GFP expression in the primary microglia (Figure 4A). When further quantitated by luciferase assays, we discovered that the transcriptional activities of these *HEXB* promoters were strong and comparable with each other (Figure 4B), further supporting the cross-species activity observed in SIM-A9 and C20 (Figure 3). Surprisingly, when tested in the primary human astrocytes, extremely low to no transcription activities were observed in the cells transfected in any species of the *HEXB* promoters (Figures 4C,D), either measured by GFP microscopy or by luciferase activity. Also, CMV promoter-driven gene transcription in astrocytes was generally much lower than in microglia but maintained at 6.77% in CMV-LG transfected cells and 41.7% in CMV-GFP transfected cells. This may be due to that internal transcription factors in astrocytes failed to efficiently support *HEXB* promoter activity even though it was transfectable as CMV promoter-driven GFP and luciferase were expressed in astrocytes. It was notable that no luciferase activities were detectable in cells transfected with CMV-driven pcDNA3-GFP plasmids because pcDNA3-GFP does not contain luciferase gene (Figures 4B,D). Like with HEK293T cells, the HexB-core promoters lacked transcriptional activity when the functional 5’-UTR was absent. Similar patterns were observed in the primary microglia and astrocytes derived from nonhuman primates, i.e., rhesus macaques (Figures 5A–D), where all these native *HEXB* promoters actively induces gene expression in microglia, but not in astrocytes. Together, these data support a notion that *HEXB* promoter is able to drive gene transcription in a microglia-specific and cross-species manner. The small-sized human *HEXB* promoter hHexB-134 may be an ideal candidate for AAV gene therapy targeting human microglia.

### Transcription Activity of *HEXB* Promoter in Comparison to Other Known Microglial Gene Promoters

**FIGURE 6:**
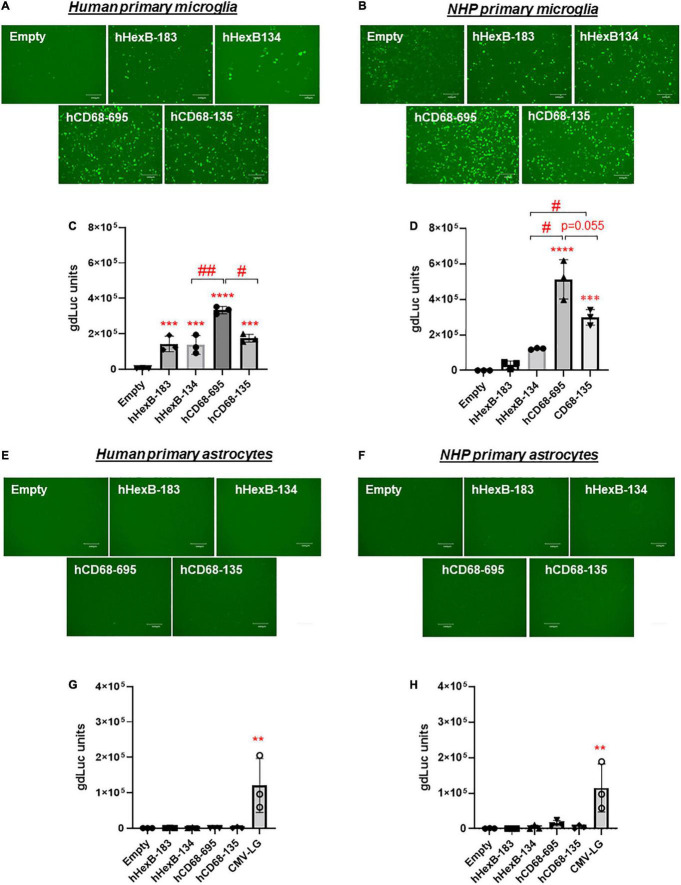
Human *HEXB* promoter-induced gene transcription in comparison to other microglia gene promoters in primary human microglia and astrocytes. Microglia-specific promoter-driven dual-LG reporters were transfected into human primary microglia **(A,C)** or human primary astrocytes **(E,G)** in 96-well plate. After transfection, GFP+ cells were observed with microscopy **(A,E)**, and gdLuc levels **(C,G)** in culture media were measured to determine the *HEXB* promoter activities. Similarly, promoter-driven dual-LG reporter were transfected into primary rhesus macaque microglia **(B,D)** or primary rhesus macaque astrocytes **(F,H)** in 96-well plate. GFP+ cells were observed with microscopy **(B,F)**, and gdLuc levels in culture media **(D,H)** were measured to determine the *HEXB* promoter activities. Scale bar, 400 μm. **p < 0.01; ***p < 0.001; ****p < 0.0001; compared with empty vector transfection controls (One-way ANOVA, *n* = 3). #*p* < 0.05; ##*p* < 0.01 (*t*-test, *n* = 3).

It has been shown that promoter from myeloid cell markers, such as CD68, were able to drive gene transcription in mouse microglia, which promotes gene targeting in microglia *via* a novel capsid-modified AAV6 vector (Rosario et al., 2016). However, it is not clear whether they have a similar promoter activity in the human microglia, particularly in the primary microglia cells. Since *HEXB* promoter displayed unique transcription activity preferred in microglia, it prompted us to compare their capacities of driving gene transcription. We cloned the well-established human *CD68* (695 bp, hCD68-695) promoter and its shorter version of 135 bp promoter (hCD68-135) (Kettenmann et al., 2011; Hickman et al., 2013; Rai et al., 2020) into our dual LG reporter vector (Figure 1). Initially, we discovered that hCD68-695 showed low transcription activity in HEK293T cells (Figure 2), but strong promoter activities in SIM-A9 and C20 cell lines (Figure 3). Surprisingly, its short version of counterpart hCD68-135 was also highly active in both microglia cell line models. Since the human *HEXB* promoter is more relevant to our human cell study, we set to examine the activities of both promoters in primary microglia. In human primary microglial cells, all these promoters were highly active in gene transcription including hCD68-695 (Figures 6A,C), which was expected. In rhesus macaque primary microglia, hHexB-183 has less promoter activity while hCD68-135 and hCD68-695 retained high transcription capacity (Figures 6B,D). When tested in the human or rhesus macaque primary astrocytes, no luciferase activity was detectable with any of these microglia-specific promoters (Figures 6E–H). The CMV-Flag LG vector served as positive transfection control (Figures 6E–H). Again, CMV-driven gene transcription in astrocytes was also lower than in microglia (Figures 6E–H). Considering the transcriptional activity and optimal size for AAV packaging, these analyses indicate that hHexB-134 promoter can be further developed to serve as a candidate microglia-specific promoter for AAV gene therapy. Our data also expanded the previous findings of CD68 promoter as an appropriate promoter by identifying a new and much short version of human CD68 (hCD68-135), ideally for gene targeting in microglia cells in the future.

## Discussion

Treatment for HIV infection has become increasingly effective in the past few decades. While antiretroviral therapy (ART) decreases viral replication to undetectable levels and improves the quality of life for those affected, there is no cure for HIV/AIDS (Eisele and Siliciano, 2012; Bender et al., 2019; Duzgunes and Konopka, 2019). Upon cessation of ART, viral loads will rebound (Eisele and Siliciano, 2012). The establishment of the latent HIV reservoirs in the brain contributes to HIV-Associated Neurocognitive Disorders (HAND), which persists in people living with HIV (PLWH) despite ART. In order to eradicate the disease as a whole, cellular HIV reservoirs hosting replication-competent HIV in the brain must be targeted by HIV cure strategies such as gene editing to excise disease-causing genetic material of HIV. The brain resident microglia act as the first line of defense against pathogens in CNS (Kettenmann et al., 2011; Wallet et al., 2019). They are long-lived (Reu et al., 2017) and can self-renew *in vivo* (Lawson et al., 1992; Ajami et al., 2007; Gomez Perdiguero et al., 2013; Bruttger et al., 2015; Askew et al., 2017; Gopinath et al., 2020). Mounting evidence supports the notion that the long-lasting HIV reservoirs establish in the CNS, where the brain microglia may constitute the major cellular CNS reservoirs (Igarashi et al., 2001; Barber et al., 2006; Churchill and Nath, 2013; Desplats et al., 2013; Avalos et al., 2017; Gama et al., 2017; Honeycutt et al., 2018; Mavigner et al., 2018; Abreu et al., 2019a,b; Wallet et al., 2019). Therefore, microglia are one of the major targets for HIV curative strategies that would permit ART-free remission and prevent CNS dysfunction. In this study, we found that the promoter of a recently characterized gene, *HEXB*, displayed a specificity in the human microglia, compared with astrocytes *in vitro*. This study is the first step for us to develop *HEXB* promoter in gene therapy for the eradication of HIV reservoirs in the brain in future.

The size of a promoter is important in gene transcription. In the initial study, mHexB-330, hHexB-183 and mkHexB-170 promoters showed the highest expression of luciferase in a transformed cell line of HEK293T. When comparing the human *HEXB* promoters of sizes 183, 134, 93, and 44 bp, the core promoters (93 and 44 bp) were ineffective in boosting luciferase expression over the promoterless vector (Empty) while the longer promoter that includes 5’-UTR was more active to induce transcription. It has been demonstrated that the essential sequences for human *HEXB* promoter activity are localized in the region between 150 and 90 bp upstream of the ATG codon (Norflus et al., 1996). The pair of promoters evaluated that contain the 5’-UTR (134 and 183 bp), had higher transcriptional activity, indicating that this 5’-UTR region is vital for human *HEXB* promoter activity. Therefore, the native promoter of *HEXB* comprising of the core promoter and the 5’-UTR is essential and minimally required to drive its transcription.

We also investigated the human *CD68* promoters. Initially, we found that 695 bp human *CD68* promoter, hCD68-695, was highly active in SIM-A9 mouse and C20 human microglial cell lines. This was similarly observed in the primary human and rhesus macaque microglia (Figures 3, 6). Surprisingly, the shorter version of the human *CD68* promoter, hCD68-135, was also active in transcription, although less active than the hCD68-695, in microglia (Figures 3, 6). In comparison to hCD68 promoters, human *HEXB* promoters, i.e., hHexB-183 and hHexB-134, strongly induced both GFP and luciferase expression in the primary human microglia, which was comparable to the short version of hCD68 promoter hCD68-135, however, slightly lower than the much longer version of hCD68 promoter hCD68-695 (Figures 6A,C).

While our data validated the previous observations of *CD68* promoter activity in mouse microglia, the size of *CD68* promoter is relatively large (695 bp), when compared to the *HEXB* promoter (134 bp). Similarly, the *F4/80* (667 bp) and *CD11b* (496 bp) promoters were shown to have some activity in AAV-vectors evaluated in microglia cells. However, these promoters are also relatively large (equal or > 500 bp) and expressed in monocyte/macrophages (Cucchiarini et al., 2003; Figures 6A,C). These observations support that HexB-135 and CD68-135 promoters warrant further investigation for microglia gene targeting. Our data support the HexB-135 promotor for its inclusion in AAV-based vectors targeted for expression in human microglia. *HEXB* is not expressed in monocytes/macrophages (Hickman et al., 2013; Masuda et al., 2020) while it might be expressed in other neural cells at a relatively low level. We do not expect the transfection of human *HEXB* promoter itself (without HEXB protein induction) will impact the expression of the other genes in the brain. More studies are needed to delineate the specific expression of *HEXB* in the human brain and the off-targets of *HEXB* promoter in the animal studies *in vivo*. We are developing novel AAV serotypes targeting human microglia for gene therapy. Also, we are constructing a new system for the inducible control of *HEXB* promoter activity in targeted microglia. These alternative approaches will further improve the targeting specificity of AAV gene therapy in the CNS.

Taken together, the *HEXB* gene promoter is a new tool suitable for microglia specific targeting of gene transfer vectors, which may be exploited for the development of gene editing therapies for the eradication of HIV reservoirs in the CNS.

## Summary

A cure for HIV would mean a lifetime free of antiretroviral therapy treatment for patients. AAV delivery of gene editors to cure HIV is promising, but the payload DNA must meet the AAV vector size limitations and express in appropriate cell types. Previous studies have identified microglia-specific mouse *Hexb* gene that shows stable expression during neural homeostasis and pathogenesis. Our study delineated the minimal/native *HEXB* gene promoter as a strong candidate for AAV gene therapy to specifically target brain microglia, the major cellular reservoirs of HIV in the central nervous system. Our studies continue to move us closer to target-specific gene therapy for NeuroHIV.

## Data Availability Statement

The original contributions presented in the study are included in the article/supplementary material, further inquiries can be directed to the corresponding authors.

## Author Contributions

WH and GJ conceived the study and wrote the manuscript with multiple revisions. SSh, KE, BB, SSa, JT, ZW, XW, and HW constructed the plasmids and performed the studies in HEK293, SIM-A9, and C20 microglia models. LW and YT performed studies involved in human and rhesus macaque primary microglia and astrocytes. BL, DM, and JG provided necessary advice during the discussion of the study. All authors read and approved the manuscript for submission.

## Conflict of Interest

The authors declare that the research was conducted in the absence of any commercial or financial relationships that could be construed as a potential conflict of interest.

## Publisher’s Note

All claims expressed in this article are solely those of the authors and do not necessarily represent those of their affiliated organizations, or those of the publisher, the editors and the reviewers. Any product that may be evaluated in this article, or claim that may be made by its manufacturer, is not guaranteed or endorsed by the publisher.

## References

[B1] Abreu C. M. Veenhuis R. T. Avalos C. R. Graham S. Parrilla D. R. Ferreira E. A. (2019a). Myeloid and CD4 T cells comprise the latent reservoir in antiretroviral therapy-suppressed SIVmac251-infected macaques. *mBio* 10:4. 10.1128/mBio.01659-19 31431552 PMC6703426

[B2] Abreu C. M. Veenhuis R. T. Avalos C. R. Graham S. Queen S. E. Shirk E. N. (2019b). Infectious virus persists in CD4(+) T cells and macrophages in antiretroviral therapy-suppressed simian immunodeficiency virus-infected macaques. *J. Virol.* 93:15. 10.1128/JVI.00065-19 31118264 PMC6639293

[B3] Ajami B. Bennett J. L. Krieger C. Tetzlaff W. Rossi F. M. (2007). Local self-renewal can sustain CNS microglia maintenance and function throughout adult life. *Nat. Neurosci.* 10 1538–1543. 10.1038/nn2014 18026097

[B4] Askew K. Li K. Olmos-Alonso A. Garcia-Moreno F. Liang Y. Richardson P. (2017). Coupled proliferation and apoptosis maintain the rapid turnover of microglia in the adult brain. *Cell Rep.* 18 391–405. 10.1016/j.celrep.2016.12.041 28076784 PMC5263237

[B5] Avalos C. R. Abreu C. M. Queen S. E. Li M. Price S. Shirk E. N. (2017). Brain macrophages in simian immunodeficiency virus-infected, antiretroviral-suppressed macaques: a functional latent reservoir. *mBio* 8:4. 10.1128/mBio.01186-17 28811349 PMC5559639

[B6] Bapat B. Ethier M. Neote K. Mahuran D. Gravel R. A. (1988). Cloning and sequence analysis of a cDNA encoding the beta-subunit of mouse beta-hexosaminidase. *FEBS Lett.* 237 191–195. 10.1016/0014-5793(88)80199-6 2971567

[B7] Barber S. A. Gama L. Dudaronek J. M. Voelker T. Tarwater P. M. Clements J. E. (2006). Mechanism for the establishment of transcriptional HIV latency in the brain in a simian immunodeficiency virus-macaque model. *J. Infect. Dis.* 193 963–970. 10.1086/500983 16518758

[B8] Bender A. M. Simonetti F. R. Kumar M. R. Fray E. J. Bruner K. M. Timmons A. E. (2019). The landscape of persistent viral genomes in ART-treated SIV, SHIV, and HIV-2 infections. *Cell Host Microbe* 26 73–85.e4. 10.1016/j.chom.2019.06.005 31295427 PMC6724192

[B9] Bruttger J. Karram K. Wortge S. Regen T. Marini F. Hoppmann N. (2015). Genetic cell ablation reveals clusters of local self-renewing microglia in the mammalian central nervous system. *Immunity* 43 92–106. 10.1016/j.immuni.2015.06.012 26163371

[B10] Churchill M. Nath A. (2013). Where does HIV hide? A focus on the central nervous system. *Curr. Opin. HIV AIDS.* 8 165–169. 10.1097/COH.0b013e32835fc601 23429501 PMC5241183

[B11] Cucchiarini M. Ren X. L. Perides G. Terwilliger E. F. (2003). Selective gene expression in brain microglia mediated *via* adeno-associated virus type 2 and type 5 vectors. *Gene Ther.* 10 657–667. 10.1038/sj.gt.3301925 12692594

[B12] Dave K. M. Ali L. Manickam D. S. (2020). Characterization of the SIM-A9 cell line as a model of activated microglia in the context of neuropathic pain. *PLoS One.* 15:e0231597. 10.1371/journal.pone.0231597 32287325 PMC7156095

[B13] Desplats P. Dumaop W. Smith D. Adame A. Everall I. Letendre S. (2013). Molecular and pathologic insights from latent HIV-1 infection in the human brain. *Neurology* 80 1415–1423. 10.1212/WNL.0b013e31828c2e9e 23486877 PMC3662272

[B14] Duzgunes N. Konopka K. (2019). Eradication of human immunodeficiency virus type-1 (HIV-1)-infected cells. *Pharmaceutics* 11:6. 10.3390/pharmaceutics11060255 31159417 PMC6631149

[B15] Eisele E. Siliciano R. F. (2012). Redefining the viral reservoirs that prevent HIV-1 eradication. *Immunity* 37 377–388. 10.1016/j.immuni.2012.08.010 22999944 PMC3963158

[B16] Gama L. Abreu C. M. Shirk E. N. Price S. L. Li M. Laird G. M. (2017). Reactivation of simian immunodeficiency virus reservoirs in the brain of virally suppressed macaques. *AIDS* 31 5–14. 10.1097/QAD.0000000000001267 27898590 PMC5131686

[B17] Gomez Perdiguero E. Schulz C. Geissmann F. (2013). Development and homeostasis of “resident” myeloid cells: the case of the microglia. *Glia* 61 112–120. 10.1002/glia.22393 22847963

[B18] Gopinath A. Collins A. Khoshbouei H. Streit W. J. (2020). Microglia and other myeloid cells in central nervous system health and disease. *J. Pharmacol. Exp. Ther.* 375 154–160. 10.1124/jpet.120.265058 32238454 PMC7569307

[B19] Hickman S. E. Kingery N. D. Ohsumi T. K. Borowsky M. L. Wang L. C. Means T. K. (2013). The microglial sensome revealed by direct RNA sequencing. *Nat. Neurosci.* 16 1896–1905. 10.1038/nn.3554 24162652 PMC3840123

[B20] Honeycutt J. B. Liao B. Nixon C. C. Cleary R. A. Thayer W. O. Birath S. L. (2018). T cells establish and maintain CNS viral infection in HIV-infected humanized mice. *J. Clin. Invest.* 128 2862–2876. 10.1172/JCI98968 29863499 PMC6026008

[B21] Igarashi T. Brown C. R. Endo Y. Buckler-White A. Plishka R. Bischofberger N. (2001). Macrophage are the principal reservoir and sustain high virus loads in rhesus macaques after the depletion of CD4+ T cells by a highly pathogenic simian immunodeficiency virus/HIV type 1 chimera (SHIV): Implications for HIV-1 infections of humans. *Proc. Natl. Acad. Sci. U.A.* 98 658–663. 10.1073/pnas.021551798 11136236 PMC14644

[B22] Kettenmann H. Hanisch U. K. Noda M. Verkhratsky A. (2011). Physiology of microglia. *Physiol Rev.* 91 461–553. 21527731 10.1152/physrev.00011.2010

[B23] Lawson L. J. Perry V. H. Gordon S. (1992). Turnover of resident microglia in the normal adult mouse brain. *Neuroscience* 48 405–415. 10.1016/0306-4522(92)90500-2 1603325

[B24] Masuda T. Amann L. Sankowski R. Staszewski O. Lenz M. (2020). P DE et al. Novel Hexb-based tools for studying microglia in the CNS. *Nat. Immunol.* 21 802–815. 10.1038/s41590-020-0707-4 32541832

[B25] Mavigner M. Habib J. Deleage C. Rosen E. Mattingly C. Bricker K. (2018). Simian immunodeficiency virus persistence in cellular and anatomic reservoirs in antiretroviral therapy-suppressed infant rhesus macaques. *J. Virol.* 92:18. 10.1128/JVI.00562-18 PMC6146711 29997216

[B26] Norflus F. Yamanaka S. Proia R. L. (1996). Promoters for the human beta-hexosaminidase genes. HEXA and HEXB. *DNA Cell Biol.* 15 89–97. 10.1089/dna.1996.15.89 8634145

[B27] Rai M. A. Hammonds J. Pujato M. Mayhew C. Roskin K. Spearman P. (2020). Comparative analysis of human microglial models for studies of HIV replication and pathogenesis. *Retrovirology* 17:35. 10.1186/s12977-020-00544-y 33213476 PMC7678224

[B28] Reu P. Khosravi A. Bernard S. Mold J. E. Salehpour M. Alkass K. (2017). The lifespan and turnover of microglia in the human brain. *Cell Rep.* 20 779–784. 10.1016/j.celrep.2017.07.004 28746864 PMC5540680

[B29] Rosario A. M. Cruz P. E. Ceballos-Diaz C. Strickland M. R. Siemienski Z. Pardo M. (2016). Microglia-specific targeting by novel capsid-modified AAV6 vectors. *Mol. Ther. Methods Clin. Dev.* 3:16026. 10.1038/mtm.2016.26 27308302 PMC4909093

[B30] Urbanelli L. Magini A. Ercolani L. Sagini K. Polchi A. Tancini B. (2014). Oncogenic H-Ras up-regulates acid beta-hexosaminidase by a mechanism dependent on the autophagy regulator TFEB. *PLoS One* 9:e89485. 10.1371/journal.pone.0089485 24586816 PMC3933543

[B31] Wallet C. De Rovere M. Van Assche J. Daouad F. De Wit S. Gautier V. (2019). Microglial cells: the main HIV-1 reservoir in the brain. *Front. Cell Infect. Microbiol.* 9:362. 10.3389/fcimb.2019.00362 31709195 PMC6821723

